# Pre-clinical models for evaluating glioma targeted immunotherapies

**DOI:** 10.3389/fimmu.2022.1092399

**Published:** 2023-01-09

**Authors:** Stephen C. Frederico, Xiaoran Zhang, Baoli Hu, Gary Kohanbash

**Affiliations:** ^1^ School of Medicine, University of Pittsburgh, Pittsburgh, PA, United States; ^2^ Department of Neurological Surgery, University of Pittsburgh, Pittsburgh, PA, United States

**Keywords:** glioma, GBM, immunotherapy, brain, tumor, pre-clinical, mouse model

## Abstract

Gliomas have an extremely poor prognosis in both adult and pediatric patient populations as these tumors are known to grow aggressively and respond poorly to standard of care treatment. Currently, treatment for gliomas involves surgical resection followed by chemoradiation therapy. However, some gliomas, such as diffuse midline glioma, have more limited treatment options such as radiotherapy alone. Even with these interventions, the prognosis for those diagnosed with a glioma remains poor. Immunotherapy is highly effective for some cancers and there is great interest in the development of effective immunotherapies for the treatment of gliomas. Clinical trials evaluating the efficacy of immunotherapies targeted to gliomas have largely failed to date, and we believe this is partially due to the poor choice in pre-clinical mouse models that are used to evaluate these immunotherapies. A key consideration in evaluating new immunotherapies is the selection of pre-clinical models that mimic the glioma-immune response in humans. Multiple pre-clinical options are currently available, each one with their own benefits and limitations. Informed selection of pre-clinical models for testing can facilitate translation of more promising immunotherapies in the clinical setting. In this review we plan to present glioma cell lines and mouse models, as well as alternatives to mouse models, that are available for pre-clinical glioma immunotherapy studies. We plan to discuss considerations of model selection that should be made for future studies as we hope this review can serve as a guide for investigators as they choose which model is best suited for their study.

## Introduction

In adults, malignant brain tumors account for approximately one-third of all CNS tumors with glioblastoma (GBM) and diffuse low-grade gliomas (LGG) being the most common subtypes (1). In children, brain tumors are the most common form of solid malignancy and account for the majority of cancer mortality (1, 2). Brainstem tumors account for 10% of all pediatric tumors within the CNS with diffuse midline glioma (DMG) being the most common subtype. The prognosis for patients diagnosed with DMG is extremely poor as greater than 90% of patients die within 2 years of their initial diagnosis (2, 3). Typical treatment of malignant gliomas involves surgical resection (in surgically accessible tumors), as well as chemotherapy and radiation therapy in lesions that are deemed higher risk (4). Unfortunately, outcomes remain poor despite this multi-modal approach and there is a dire need for new therapeutic modalities (4–6).

Neoplastic cells are constantly generated throughout a person’s lifetime, most of which are inevitably removed by the host immune system through anti-tumor immunity. The few neoplastic cells that manage to escape anti-tumor immunity eventually become a tumor (7). The concept of immunotherapy is the promotion of immune recognition, activation, and elimination of neoplastic cells. Immunotherapy in the form of immune checkpoint inhibitors (ICI) have radically transformed the treatment paradigm of cancer. ICIs are able to induce dramatic and durable response in many solid tumors and have now become the first-line treatment for the treatment of melanoma, colorectal cancer, and non-small cell lung cancer (8). Other immunotherapy approaches include adoptive cell transfer, cytokine/chemokine-based therapies, and tumor vaccination. Given the lack of effective therapies in malignant gliomas and the effectiveness of immunotherapy for other solid malignancies, immunotherapy for malignant gliomas has become an area of great interest.

Pre-clinical studies in animal models of malignant gliomas have yielded many promising immunotherapy candidates, many of which have eventually failed in clinical trials (9, 10). This discrepancy between pre-clinical and clinical results, points to the failure of pre-clinical models of malignant gliomas at recapitulating the tumor immune cell interactions within the tumor microenvironment. Many pre-clinical options are currently available, each one with their own benefits and limitations. Informed selection of pre-clinical models for testing can facilitate translation of more promising immunotherapies in the clinical setting. Here, we review commonly used and recently developed glioma cell lines, mouse models, as well as alternative animal models, in an effort to highlight which of these may be best suited for immunotherapy studies.

## Key considerations for pre-clinical models of glioma

Immunotherapy is a catch-all term that includes a wide variety of approaches of manipulating the host immune system to eliminate cancer. As such, there is no one perfect pre-clinical model to evaluate the different immunotherapy approaches in gliomas. We propose several key considerations that investigators should take when selecting pre-clinical models of glioma for evaluation of immunotherapy.

### Tumor origin

The first and perhaps the most impactful decision the investigator has to make is the origin of the tumor. They can be from the same species as the model animal (allogeneic) or patient-derived (xenograft). Allogeneic tumors can be generated from spontaneously occurring tumors, carcinogen mutagenesis, genetic engineering, and transposon mutagenesis. Xenograft tumors are patient-derived cell lines and cancer stem cells (CSCs). Allogeneic tumors can be implanted on immunocompetent mice whereas xenograft models can only be implanted in immunocompromised or humanized mice.

### Tumor antigen expression

Therapeutic approaches such as CAR-T and tumor vaccines require that animal models express some of the same tumor neo-antigens as the human tumor. In this respect, genetically engineered mouse models (GEMMs) of gliomas are not always the best choice. GEMM does a good job at recapitulating driver mutations, however, these tumors poorly express tumor neoantigens that are expressed by gliomas, limiting the usefulness of this model when evaluating immune therapies.

### Tumor mutational burden

For some tumors residing outside the CNS, it has been observed that tumors having a higher mutational burden are better candidates for immunotherapy. This higher mutational burden often leads to the production of more tumor neoantigens which can be targeted by the immune system. This observation has been made in colorectal cancer, as well as other cancers outside the CNS (11, 12). However, the opposite has been observed in gliomas, where a higher tumor mutational burden is often associated with worse survival (12, 13). These findings highlight the need for paying close attention to tumor mutational burden when choosing a pre-clinical model for glioma immunotherapy studies as models having very high tumor mutational burden may not be best suited for immunotherapy studies.

### Growth rate

Gliomas are known as aggressive malignancies that are known to grow quickly. It has been reported that GBM specifically has a median specific growth rate of 1.4% per day, with an equivalent volume doubling time of 49.6 days (14). Choosing a pre-clinical model that has a high growth rate is crucial for immunotherapy studies as these tumors grow quickly in patients. Additionally, GBM as well as other gliomas, grow in an infiltrative manner unlike most CNS tumors (15). Given these findings, it is crucial for investigators performing glioma immunotherapy studies to choose pre-clinical models that possess high growth rates and closely parallel glioma growth patterns as this will best replicate what is observed in patients.

## Cell lines and mouse models of glioma

### GL261

GL261 is an allogeneic tumor cell line that was originally created by intracranially injecting C57BL/6 mice with a known carcinogen, that being, 3-methylcholantrene (16). Small pieces of the tumor were taken and subjected to serial passaging over time which is believed to be one of the reasons that GL261 lacks important glial differentiation markers (17). The growth of intracranial GL261 tumors has been described in the literature as rapid with a slightly invasive growth pattern. Additionally, it has been noted that lymphocyte infiltration is extremely low in these tumors. Szatmári and colleagues found that after intracranially implanting 1 × 10^5^, 1 × 10^4^, 1 × 10^3^ and 1 × 10^2^ GL261 cells into immunocompetent mice, the mean survival time was 25, 27, 36 and 55 days respectively (16). It has been noted that a higher level of MHC1 antigens can be detected in wildtype GL261 tumors when compared to healthy brain, and it has been noted that MHC1 is upregulated in cells exposed to interferon-gamma (16). Compared to other tumor lines, GL261 has a higher mutational burden as whole exome sequencing has shown *in vitro* GL261 to have 212 frameshift and 4766 missense mutations (18). In the same study, it was shown that *in vitro* SB28 had 67 frameshift and 41 missense mutations (18). Commonly, GL261 cells are administered to mice *via* intracranial injection, but these tumors can also be grown in the subcutaneous space by injecting mice with GL261 cells in the flank.

### SMA-560

After H. Fraser and colleagues observed the first incidences of mice developing spontaneous gliomas, Serano and colleagues developed the SMA-560 cell line after performing a serial transplantation of spontaneous murine astrocytoma (19). Specifically, tumor tissue underwent homogenization, *in vitro* culturing, and subsequent transplantation into VM/Dk mice (19). The median survival for animals bearing SMA-560 tumors following injection with 1×10^4^ tumor cells/5 μl has been reported to be approximately 26 days (20). Notably, SMA-560 has high expression of glial fibrillary acid protein (GFAP) and the astrocyte marker glutamine synthetase, and low expression of S-100 proteins (21, 22). Additionally, it has been noted that while MHC1 expression is low in SMA-560 at baseline, it is upregulated in cells exposed to interferon-gamma (23). In a study by Johanns and colleagues, it was observed that SMA-560 had 2171 non-synonymous exome mutations as compared to 4,932 for GL261 (24). SMA-560 cells can be administered to mice *via* intracranial injection, but these tumors can also be grown in the subcutaneous space by injecting mice with SMA-560 cells in the flank.

### CT-2A

CT-2A is an allogeneic cell line that was generated from a malignant astrocytoma that was formed in C57BL/6J mice that were injected in the cerebrum with a known carcinogen, that being, 20-methylcholanthrene (25). These cells have a high tumorigenicity as mice have a median survival of 20 days after intracranial injection with 1x10^4^ cells (23). This tumor is known to have high levels of complex gangliosides and very low distribution of GM3 (monosialodihexosylganglioside) which has been classified as an anti-angiogenic ganglioside (26, 27). Additionally, CT-2A cells are known to be deficient in the tumor suppressor PTEN and are wild-type for p53. These tumors have a high mitotic index and unlike many other tumors, demonstrate high levels of microvascular proliferation (25, 28). Commonly, CT-2A cells are administered to mice *via* intracranial injection, but these tumors can also be grown in the subcutaneous space by injecting mice with CT-2A cells in the flank.

### SB-28

SB28 is an allogeneic cell line that was generated by using sleeping beauty transposons to insert constructs capable of targeting the P53, RAS and PDGF pathways. These sleeping beauty transposon flanked pT2/CAG-NRasV12 and pT2/shp53/mPDGF constructs were then injected into the right ventricle of C57L/6 mice (23). It has been noted that SB28 bearing mice have extremely low MHC1 expression and limited CD8 T cell infiltration posing a challenge for immunotherapy-based studies using this cell line (18). The median overall survival for mice injected with 1x10^4^ SB28 cells is 19 days and whole exome sequencing has demonstrated these cells have just 108 mutations as compared to the over 4900 mutations present in GL261 (18, 24). This cell line can be injected intracranially, but these tumors can also be grown in the subcutaneous space by injecting mice with SB28 cells in the flank.

### U251

U251 is a xenograft cell line that was derived from a glioblastoma multiforme using explant technique (29). These cells must be injected into immunocompromised mice and the median overall survival for tumor bearing mice is 22 days (30). It has been noted in previous studies that B7-H4 expression is upregulated in U251 glioma stem-like cells and while U251 cells do not carry an IDH1 mutation, these cells do carry mutations in hTERT, PTEN and p53 (31). Additionally, these cells have a methylated MGMT status (31). These cells must be injected into immunocompromised mice, limiting their usability in immunotherapy-based studies.

### U87

U87 is a xenograft cell line that was derived from a GBM in a female patient. Immunocompromised mice bearing U87 tumors have a median survival of 28.6 days following tumor implantation (30). Interestingly, it has been observed that U87 and U251 tumors only grow to kill their hosts when 1,000,000 or 1,500,000 cells, respectively, are injected in the striatum of nude mice (30). It has been shown that injecting less cells leads to a lack of tumor growth and avoidance of death of the host. It has been noted that U87 cells possess hTERT, ATRX and PTEN mutations, however, these cells do not carry p53 or IDH1 mutations (31). Additionally, these cells have a methylated MGMT status (32). These cells must be injected into immunocompromised mice and can be injected intracranially or into the flank region.

### Qk/Trp53/PTEN (QPP) Triple-knockout glioma model

QPP is an immunocompetent murine spontaneous GBM model, in which three common patient-relevant tumor suppressor genes, Quaking (Qk in mouse and QKI in human), Trp53, and PTEN, were deleted (33). The tumors that were derived from this model displayed histopathological and transcriptomic heterogeneity, which can manifest the subtypes of GBM (33). The cell line QPP7, isolated from this model, was used to establish the syngeneic orthotopic glioma in C57L/6 mice with genetic manipulations in previous research (34). Importantly, this syngeneic mouse glioma demonstrated the landscape of the tumor immune microenvironment, including M1/M2-like macrophages, T cells, NK cells, and myeloid-derived suppressor cells (MDSCs) (34). Based on immune profiling and single-cell sequencing analyses, a most recent study reported that both implanted and spontaneous QPP models recapitulate the immunosuppressive myeloid dominant nature of the tumor microenvironment of human gliomas (35).

### Cre-LoxP transgenic glioblastoma mouse model

The Cre-LoxP system allows for the targeting of tumor genes in mouse brain tissue of interest which provides for insight into the genetic drivers of GBM and the differences in genetic drivers between primary and secondary GBMs (36). To create this model, a mouse strain known as the “Cre driver strain” which has Cre recombinase with a promoter, and a mouse strain known as the “LoxP floxed strain” that has LoxP floxed exons of the target gene, were bred together (36). By breeding these strains together this would result in a deletion of the floxed region and a subsequent inactivation of the gene in the desired brain tissue of interest, leaving the target gene active in tissues outside this region (36). Specifically, this model has been used to test the role of p53 and PTEN function in GFAP positive GBM. In a study by Zheng and colleagues, the research team created a p53 and PTEN double knockout mouse where this knockout was targeted to astrocytes specifically (37). The research team found that a loss of both p53 and PTEN would regulate Myc levels and subsequently control NSC self-renewal and differentiation (37, 38). The Cre-LoxP mouse model is extremely valuable for testing immunotherapy applications as this model can activate or inactivate genes that can impact the tumor microenvironment, and this system has also been used to control the cytotoxic potential of CAR-T cells (39).

### Humanized glioma mouse model (HGMM)

The HGMM model was developed by Huang and colleagues to better understand the role of CCL18 which is expressed in humans but not in rodents (40). Specifically, the research team developed this model to study the interaction of human glioma cells with human microglia. To create this model, the research team depleted intrinsic microglia from murine organotypic brain slices and then injected either human glioma cells into these slices, or injected both human glioma cells and human stem cell derived microglial cells into these slices (40). Interestingly, the research team found that in slices injected with both human glioma cells and human stem cell derived microglial cells, human stem cell derived microglial cells showed higher levels of sphericity and cell body volume highlighting how the tumor microenvironment impacts the morphology of these cells (40). Additionally, the research team assessed whether the presence of human stem cell derived microglial cells with human glioma cells created an increase in the expression level of genes known to be upregulated by the glioma environment. The research team found that there was an upregulation in IL-10, Osteopontin, MMP14, VEGF, TGF β, and CCL18 in samples containing both human stem cell derived microglial cells with human glioma cells, as opposed to samples containing human glioma cells alone (40).

Ultimately, this model highlights that the presence of human stem cell derived microglial cells with human glioma cells results in not only larger tumors but also an upregulation of genes similar to those known to be upregulated in certain gliomas (40–45). Recent findings have begun to shed light on how GBM can use microglia to induce immunosuppression within the tumor microenvironment. This model will be valuable in developing a deeper understanding of this hijacking and potentially enable therapeutic exploitation of this mechanism (46).

## Alternative animal models of glioma

While glioma mouse models are beneficial to use in immunotherapy studies due to their low cost and availability, these models certainly have their limitations (47–49). Some of the limitations of mouse models include the lack of a highly immunosuppressive glioma microenvironment that is commonly observed in human gliomas, and that patient derived xenografts often must be transplanted into immunocompromised rodents (47, 48, 50). These limitations along with others highlight the need for creating alternative models that can be used in pre-clinical glioma immunotherapy studies.

Drosophila melanogaster is one alternative to glioma mouse models as 75% of human genes share functional orthologs with drosophila (50, 51). This finding makes drosophila a useful model for studying gliomagenesis as gliomas can be induced in this model using the GAL4/upstream activation sequence system (50, 52, 53). Additionally, this model is valuable for studying centromere dysfunction which has been shown to lead to tumor development as a result of perturbation of stem cell division (50, 54). However, it is important to note that this model lacks an adaptive immune system and relies on humoral and cell-mediated innate immunity for its defense against pathogens, limiting this models role in immunotherapy-based studies (55–57).

Canine brain tumor models are an exciting large animal model that have recently been developed for neuro-oncology studies (58). It has been demonstrated that intracranial gliomas spontaneously arise in canines and that these tumors share similar morphological and immunological characteristics with human gliomas (50, 59). Additionally, molecular characterization of canine gliomas has shown that these tumor share similar somatic alterations that are known drivers of human gliomas such as mutations in Tp53 and IDH (60–62). Immunotherapy studies in glioma-bearing canines are limited at this time as this model is still new to the neuro-oncology space and a limited number of canines develop gliomas on an annual basis (63). However, the limited number of studies that have occurred in glioma-bearing canines have highlighted the promise associated with this model (63–66).

Danio rerio (zebrafish) is a final alternative model that should be considered for glioma immunotherapy studies as zebrafish lack an adaptive immune system until six weeks of age, allowing for the implantation of human glioma cells that lead to an invasive glioma (50, 67, 68). Additionally, this model has a similar microenvironment with regards to density, to what is observed in the human brain (50). Limitations of this model include differences in the tumor microenvironment compared to that of humans, and that the optimal temperature for human cells is 37°C compared to fish cells which is 28°C (50, 69). Recently, a zebrafish model has been developed that can engraft human tumors at 37°C (70). It will be interesting to observe whether this model can be used in future immunotherapy studies.

## Conclusion

In this manuscript we present glioma cell lines and mouse models, as well as alternative glioma animal models that can be used in immunotherapy studies. Additionally, we discuss some of the benefits and limitations associated with these animal models (see **Table 1**). When choosing a model we believe it is first crucial to assess where the tumor is derived from. Specifically, some allogeneic models such as GL261, CT2A, etc. were created by administering known carcinogens to mice, which is believed to be dissimilar to how gliomas arise within humans. Models created *via* carcinogens do not replicate the developmental biology of gliomas to the fullest extent and as such may not be best suited for immunotherapy studies as these models do not entirely possess the immunosuppressive mechanisms observed in human gliomas (9, 10, 91).

**Table 1:** Benefits and limitations associated with animal models of glioma.

Glioma Models	Benefits	Limitations	References
Mouse	• Affordability • Many murine glioma cell lines are available and can be implanted into this model	• Tumor growth patterns are dissimilar to what is observed in humans • Glioma microenvironment is dissimilar to what is observed in humans • Patient-derived cell lines must be implanted into immunocompromised mice • Many murine glioma cell lines and patient derived xenograft mouse models were generated in ways that are believed to be dissimilar to how human gliomas develop (carcinogen use, etc.) • Many murine glioma cell lines have more antigen expression than what is observed in human gliomas	(47–50, 71–82)
Drosophila Melanogaster	• Affordability • Share many similar genes with humans as 75% of human genes share functional orthologs with drosophila • Excellent models for studying brain tumor development	• Lack an adaptive immune system, limiting this model’s value in immunotherapy-based studies	(50–57, 83–87)
Canine	• Spontaneously develop gliomas that share similar morphological and immunological characteristics with human gliomas • Canine gliomas share similar somatic alterations with human gliomas that are known drivers of human glioma development	• An expensive model that requires advanced animal care facilities with a trained veterinary staff • Limited glioma immunotherapy studies have been performed in this model • A limited number of canines develop brain tumors on an annual basis which limits the number of canine based studies that can occur	(50, 58–63, 88, 89)
Danio Rerio (Zebrafish)	• Affordability • Can implant human glioma cells into this model prior to it generating an adaptive immune system	• The brain is more difficult to image with age as optical transparency is ultimately lost • The glioma microenvironment is dissimilar to what is observed in humans • Limited glioma immunotherapy studies have been performed in this model	(50, 67–70, 90)

Tumor antigen expression and mutational burden are also important considerations when choosing a brain tumor model for pre-clinical brain tumor immunotherapy studies. Many gliomas, especially GBM and DMG, are known as “immunologically cold” tumors as these malignancies express few antigens and have low mutational burdens. Many allogeneic mouse models and some patient derived xenografts such as U87 have increased antigen expression and/or tumor mutational burden. This is problematic for pre-clinical immunotherapy studies as findings showing efficacy in these pre-clinical models may largely be due to there being more antigenic targets than there should be and/or an increased mutational burden, suggesting that a therapy is fit for clinical trial when it truly is not. Canine and zebrafish models may be beneficial in future glioma immunotherapy studies as canines spontaneously generate gliomas, and zebrafish can undergo transplantation with patient derived xenografts prior to their generation of an adaptive immune system. Ultimately however, we believe that selecting a model that is both patient derived, and “immunologically cold” is crucial for future pre-clinical glioma immunotherapy studies as we believe this will help reduce the number of failed immunotherapy clinical trials observed in the neuro-oncology space.

## Author contributions

SF, XZ, and GK contributed to the conception and design of the review. The first draft was written by SF, XZ, and BH. GK supervised the writing of the manuscript and is the corresponding author. All authors contributed to the manuscript and approved the submitted version.
